# Piroplasm infestations in cattle: exploring tick control using *Chrysanthemum* extract and neem oil emulsion

**DOI:** 10.3389/fvets.2025.1543162

**Published:** 2025-03-31

**Authors:** Salwa Mahmoud Abd-Elrahman, Fatma Atea Kamel, Sara Salah Abdel-Hakeem, Abeer A. Khedr, Shaymaa M. Mohamed, Ahmed A. Abdelgaber, Madeha Darwish, Ahmed M. Al-Hakami, Abdulah J. Alqahtani, Ahmed Kamal Dyab

**Affiliations:** ^1^Department of Parasitology, Faculty of Veterinary Medicine, Assiut University, Assiut, Egypt; ^2^Parasitology Laboratory, Department of Zoology and Entomology, Faculty of Science, Assiut University, Assiut, Egypt; ^3^Department of Biosciences, Durham University, Durham, United Kingdom; ^4^Department of Parasitology, Faculty of Veterinary Medicine, New Valley University, El-Khargah, Egypt; ^5^Department of Pharmacognosy, Faculty of Pharmacy, Assiut University, Assiut, Egypt; ^6^Department of Pharmacognosy and Pharmaceutical Chemistry, College of Pharmacy, Taibah University, Medina, Saudi Arabia; ^7^Department of Animal, Poultry, and Aquatic Life Behavior and Management, Faculty of Veterinary Medicine, Assiut University, Assiut, Egypt; ^8^Department of Animal and Poultry Behavior and Management, Faculty of Veterinary Medicine, Assiut University, Assiut, Egypt; ^9^Department of Clinical Microbiology and Parasitology, College of Medicine, King Khalid University, Abha, Saudi Arabia; ^10^Department of Microbiology and Clinical Parasitology, College of Medicine, King Khalid University, Abha, Saudi Arabia; ^11^Department of Medical Parasitology, Faculty of Medicine, Assiut University, Assiut, Egypt; ^12^Department of Parasitology, School of Veterinary Medicine, Badr University in Assiut, Assiut, Egypt

**Keywords:** *Theileria annulata*, *Babesia bigemina*, *Rhipicephalus annulatus*, hemolymph, acaricides, scanning electron microscope

## Abstract

**Introduction:**

Tick-borne diseases represent a major threat to both animal and human health globally. This study explores the prevalence of tick infestation and associated piroplasm infections specifically *Theileria* and *Babesia* species in cattle, in addition to evaluating the acaricidal effectiveness of *Chrysanthemum* extract (*Dendranthema grandiflora*) and neem oil emulsion (*Azadirachta indica*).

**Methods:**

Among 130 cattle examined, 61 were infested with ticks and subsequently screened for piroplasm infections. Molecular analysis identified infections caused by *Theileria annulata* and *Babesia bigemina*.

**Results:**

A strong association was found between tick infestation and *Babesia* species, while *T. annulata* infection showed a slight correlation. Hemolymph examination confirmed the critical role of ticks in the life cycle of piroplasm infection. *Chrysanthemum* extract and neem oil were tested for their acaricidal properties against adult ticks (*Rhipicephalus annulatus*). *Chrysanthemum* extract (0.5 mg/mL) caused tick mortality within 24 h. However, neem oil induced rapid and significant tick mortality at (20 mg/L) and (15 mg/L), achieving 100% mortality within the same time frame. Both treatments demonstrated high effectiveness, with results indicating strong dose-and time-dependent effects compared to controls. Scanning electron microscopy (SEM) revealed extensive morphological damage to treated ticks. This damage included destruction of the hypostome, loss of surface striations, wrinkling with pore formation, and cracking following exposure to neem oil and *Chrysanthemum* extract.

**Discussion:**

These findings highlight the potential of *D. grandiflora* extract and neem oil emulsion as effective natural acaricides for controlling tick infestations and reducing tick-borne diseases.

## 1 Introduction

Ticks are blood-feeding ectoparasites that pose a global health concern. The cattle tick, *Rhipicephalus annulatus*, is one of the most economically significant vectors affecting human and animal health (1). Distributed across many tropical and subtropical areas, where they naturally contribute to the maintenance of tick-borne diseases in both animals and humans (2). Consequently, ticks may be an effective indicator for tracking tick-borne parasites (TBPs) due to their capacity to transmit blood and pathogens from many hosts. Data on the geographical distribution of pathogens in ticks help assess the risk of exposure to tick bites and, consequently, the risk of disease transmission (3).

Tick-borne diseases such as theileriosis and babesiosis significantly threaten the cattle industry (4). Blood parasites are transmitted when infected ticks inject parasites into the host’s bloodstream while feeding (5). In Egypt, *T. annulata* causes tropical theileriosis, characterized by symptoms such as fever, anorexia, jaundice, tachycardia, difficulty breathing, lymphadenopathy, and general weakness, severely impacting cattle productivity (6). Different species of *Babesia*, (*B. divergens*, *B. naoakii*, *B. bigemina*, and *B. bovis*) cause babesiosis, which is characterized by fever, hemolytic anemia complicated with neurological and respiratory disorders, which can be fatal (7). The molecular characterization and phylogenetic analysis of *Babesia* and *Theileria* are essential for understanding the diversity, evolution, and epidemiology of these protozoan parasites (28). This knowledge is crucial for developing targeted control measures, vaccines, and diagnostic tools, which ultimately aid in managing tick-borne diseases. Additionally, genetic characterization can provide insights into host-parasite interactions and help predict how these pathogens may adapt to environmental changes or develop resistance to treatments. This underscores the importance of this research in both veterinary and human health (59).

Therefore, addressing tick control is essential for sustaining cattle health and productivity (8). Currently, the National Drug Authority has registered more than 25 acaricide brands readily available to farmers (9). Regrettably, ticks in Egypt have developed resistance to all available acaricides (10). This resistance and environmental and health concerns have driven a shift toward exploring alternative tick control methods (11). Consequently, there is upwards interest in evaluating the acaricidal efficacy of natural, environmentally sustainable, and safer biological agents, such as essential oils and plant extracts (12).

Natural products often contain bioactive compounds that exhibit insecticidal, repellent, and growth-regulating properties (13). Among these alternatives is Pyrethrum, which is recognized as a substantial reservoir of pyrethrins (14). They are derived from the flowers of *Chrysanthemum cinerariaefolium* and have been used as insecticides for millennia. These natural neurotoxins target the nervous system of insects, causing paralysis and death (15). They are effective against a broad spectrum of pests as they act rapidly upon contact (16). The development of synthetic pyrethroids, such as permethrin, has enhanced the insecticidal properties and environmental stability of these compounds. Consequently, they are extensively utilized in agricultural, veterinary, and household pest control products (17). Other species of *Chrysanthemum* may have insecticidal properties for tick control, but there is limited research on their effectiveness against *R. annulatus* ticks.

Neem oil, derived from the seeds of the neem tree (*Azadirachta indica*), is a natural source of insecticidal compounds. It contains a complex mixture of bioactive compounds. The primary compound, azadirachtin, disrupts various physiological processes in ticks, such as feeding, reproduction, and molting (18, 19). It acts as an insect growth regulator (IGR) and interferes with the synthesis and release of molting hormones, leading to disruption of the molting process and future reproduction (20). Additionally, azadirachtin reduces ticks by deterring them from attaching to and feeding on their hosts due to its anti-feedant and repellent properties. Using botanical insecticides, such as *Chrysanthemum* extract and neem oil, is consistent with the principles of integrated pest management (IPM). IPM underscores the use of multiple, complementary control strategies to attain effective acaricidal management while reducing its environmental impact (21). Despite the potential of *Chrysanthemum* extract and neem oil as tick control agents, significant knowledge gaps need addressing. Research is needed to determine the best formulations, application methods, and dosages for these insecticides to maximize their effectiveness in controlling *R. annulatus* while ensuring the safety of animals. This research aims to provide valuable insights for the development of alternative and eco-friendly strategies for tick control.

## 2 Materials and methods

### 2.1 Sample collection and study area

A total of 130 cattle, aged between one and five years old, were examined at the veterinary clinic of the Faculty of Veterinary Medicine at Assiut University in Assiut, Egypt, from January 2023 to December 2023. The examined cattle consisted of 52 males and 78 females. Only infested animals with ticks (61) were included in the study to further assess tick-piroplasm association.

### 2.2 Blood collection and thin blood film examination

Five milliliters of blood were collected from the jugular vein of all animals using sterile vacutainer tubes containing anticoagulants. The blood samples were then transported on ice to the Parasitology Department for further examination. Fresh thin blood smears were prepared, dried, and fixed in methanol to identify piroplasm infection using a light microscope (Olympus BX43F, Tokyo 163-0914, Japan). Positive samples were used for molecular analysis and species identification (22).

### 2.3 Genetic characterization of *Theileria* and *Babesia* species

#### 2.3.1 DNA extraction and PCR amplification

All positive samples with same morphological characteristics, obtained from the same host and region, underwent DNA genome extraction using the QIAamp DNA Mini Kit (Catalogue 51304, Qiagen). Two target genes were selected for amplification: *tams1* for *Theileria annulata* and 18S rRNA for *Babesia*. The primer sequences for each gene were as follows: *tams1* primers were 5′-GTAACCTTTAAAAACGT-3′ and 5′-GTTACGAACATGGGTTT-3′, yielding a 721 bp product (23). The 18S rRNA primers were 5′-GTCTTGTAATTGGAATGATGGTGAC-3′ and 5′-ATGCCCCCAACCGTTCCTATTA-3′, producing a 340 bp product (24). PCR amplification was carried out using the EmeraldAmp GT PCR Master Mix (Takara, Code No. RR310A). Each reaction mixture involved (12.5 μL) of 2× premix, (5.5 μL) of PCR-grade water, (1 μL) of each primer (20 pmol), and (5 μL) of a DNA template, and the total reaction volume was (25 μL). The conditions of the thermal cycling started with initial denaturation for 5 min at 94°C, followed by 35 cycles for 30 s at 94°C, annealing for 40 s at 55°C, and extension for 45 s at 72°C (for *tams1*), and 40 s (for 18S rRNA), final extension for 10 min at 72°C end the PCR. Ten microliters aliquots of each PCR reaction were loaded onto a 1.5% agarose gel in a horizontal gel electrophoresis system (Compact M, Biometric, Germany), stained with ethidium bromide, and visualized under UV light to confirm successful amplification (25).

#### 2.3.2 Purification of PCR products

The QIAquick PCR Purification Kit (Qiagen Inc., Valencia, CA) was used to purify PCR products. The PCR product was mixed with Buffer PB1 (five volumes), the mixture was added to a QIAquick spin column, washed with Buffer PE, and then the purified DNA was eluted with nuclease-free water. This procedure needs approximately 10 min to produce purified DNA suitable for subsequent sequencing applications.

#### 2.3.3 DNA sequencing and analysis

PCR products were sequenced in forward and reverse directions using an automated DNA sequencer (Applied Biosystems, United States). The sequencing was performed with the BigDye Terminator v3.1 Cycle Sequencing Kit (Perkin-Elmer/Applied Biosystems). After sequencing, the NCBI BLAST tool was used to compare the sequences to those in GenBank that already existed. This comparison allowed us to identify similarities and differences between the freshly generated sequences and those already in the database.

### 2.4 Phylogenetic analysis

Following sequencing, the obtained sequences were aligned using the CLUSTAL W algorithm, integrated into the MegAlign software (version 12.1) (26). MEGA11 software was used to perform Phylogenetic analyses, engaging multiple methods such as maximum likelihood, neighbor-joining, and maximum parsimony (27).

### 2.5 Ticks’ isolation and identification

The ticks were carefully removed using soft forceps and prepared for morphological characterization. They were steeped in potassium hydroxide solution (5%) and then transferred to 2.5% an acid alcohol solution for pH regulation, dehydrated in an escalating sequence of ethanol alcohol, and finally immersed in xylene to become transparent for 24 h (28). Microscopic and molecular identification of tick’s species, *Rhipicephalus annulatus*, was previously reported using the *coxI* gene and deposited in the Gene bank under accession number (OR965090) (28).

### 2.6 Direct examination of hemolymph

The isolated ticks were used to prepare the hemolymph smears, to detect the presence of ookinetes of piroplasm following the method defined by Allam et al. (29). The procedure involved amputating the distal portion of one or more legs of the tick to obtain a small drop of hemolymph on a clean glass slide. The smears were air dried, fixed in absolute methanol for 10–15 min, stained with Giemsa’s stain, and examined under an oil immersion light microscope.

### 2.7 Preparation of *Chrysanthemum* extract

In May 2024, the aerial parts of the *Chrysanthemum* (*Dendranthema grandiflora*) were collected from the Floriculture Farm at the Faculty of Agriculture, Assiut University, Egypt. *Chrysanthemum* is an herbaceous flowering plant known for its ornamental significance. It encompasses a range of varieties displaying notable genetic diversity in traits such as flower yield, quality, and growth parameters. The specific variety under investigation is characterized by its white flowers. The plant material was air-dried, ground into a 200 g powder, and macerated in 70% methanol for 24 h. After filtration and concentration using a rotary evaporator, this process was repeated to obtain a 60 g dry residue. The *Chrysanthemum* extract was then diluted in distilled water and glycerol to create a series of varying concentrations: 0.125, 0.25, and 0.5 g/mL (30).

### 2.8 Determination of total phenolic acid of *Chrysanthemum* extract

In the investigation of total phenolic acid levels, a stock solution of gallic acid was prepared in methanol at a concentration of 2 mg/mL. A series of dilutions were prepared from the stock solution to achieve concentrations of 1,000, 750, 500, 375, 250, 187.5, and 125 μg/mL. Additionally, a *Chrysanthemum* extract was prepared in methanol at a concentration of 10 mg/mL. Folin–Ciocalteu method was used to quantify the total phenolic content, succeeding the procedure described by Kamtekar et al. (31). Briefly, 10 μL of either the sample or standard solution was mixed with 100 μL of Folin–Ciocalteu reagent (1:10) in a 96-well microplate. After mixing, 80 μL of 1 M Na_2_CO_3_ was added to the mixture and incubated at 25°C for 20 min in the dark. The resulting blue color complex was quantified at 630 nm using a spectrophotometer. The collected data were analyzed and presented as means ± SD. We used a FluoStar Omega microplate reader to record the results.

### 2.9 Determining the total flavonoid content of *Chrysanthemum* extract

For this purpose, the stock solution of standard rutin was prepared in methanol (CH_3_OH) at a concentration of 2,000 μg/mL. A series of dilutions were then made from this stock solution to achieve concentrations of 1,000, 500, 250, 125, 62.5, 31.25, and 15.625 μg/mL. Furthermore, the *Chrysanthemum* extract was prepared in CH_3_OH at a concentration of 10 mg/mL. The total flavonoid content was evaluated using the AlCl_3_ colorimetric assay as mentioned by Nurcholis et al. (32). For the assay, 15 μL aliquots of both the sample and standard rutin solutions were dispensed into a 96-well microplate. Then, 175 μL of CH_3_OH, 30 μL of 1.25% AlCl_3_, and 30 μL of 0.125 M CH_3_COONa were sequentially added. The mix was then incubated at room temperature for 5 min, the yellow color mix was measured at 420 nm using a UV/VIS spectrophotometer. The data are presented as means ± standard deviation (SD). The results were recorded using a FluoStar Omega microplate reader.

### 2.10 Preparation of neem oil emulsion

Neem oil was obtained from Centa Kind Pharmaceuticals (RC# 7797, Nefertari, Limited Co., El-Fayoum, Egypt). This oil was derived from the seeds of the neem tree (*Azadirachta indica*, family: Meliaceae) and is known for its high purity level of over 98%. Commercial formulations often contain standardized concentrations of active ingredients, such as azadirachtin, which enhances their effectiveness. This standardization ensures that the product delivers reliable results, making it preferable for therapeutic applications. To prepare the emulsion, the neem oil was combined with the non-ionic surfactants (soap) in a 1:3 ratio. The process began by mixing deionized water and surfactants using a stirrer, followed by the addition of neem oil. Neem oil concentrations of 10, 20, and 25% were prepared according to Gareh et al. (33).

### 2.11 Acaricidal potential of *Chrysanthemum* extract and neem oil on *Rhipicephalus annulatus* adult stage

The *in vitro* adult immersion test (AIT) technique was used to evaluate acaricides sensitivity assay and water with four replications as described by FAO (34). Briefly, in sterile petri dishes, three concentrations were prepared from *Chrysanthemum* extract (0.5, 0.25, 0.125 mg/mL) and neem oil (20, 15, 10 mg/L). Phoxim (1 mL/L) was used as a positive control, deionized water, and Tween 80% (2:1) were used as the control negative group. Ten moderately engorged females (nearly the same size) were placed in 3 mL of each concentration from each treatment for 5 min. In sterile 6-well plates with filter paper, three replicates for each concentration were used to incubate the treated ticks at 28°C with a relative humidity of (80 ± 5%). Later, ticks were observed at various time intervals (3, 6, 12, 24, 48, and 72 h). Viability checks were regularly performed, if a tick did not respond to needle stimulation, it was considered dead. The mortality rate was calculated using the equation below:


$$\begin{array}{c} \mathrm{Mortality rate}\;\left(\%\right)=\left(\begin{array}{l} \mathrm{Mortality rate in treated group}\\ -\mathrm{Mortality rate in control group} \end{array}\right)/\\ \left(100-\mathrm{Mortality rate in control group}\right)\times 100. \end{array}$$

### 2.12 Evaluation of the acaricidal effect using scanning electron microscopy

*R. annulatus* adult ticks from the treated groups with neem oil (20 mg/L), *Chrysanthemum* (5 mg/L), phoxim (1 mL/L), and control negative were selected and preserved in 2.5% glutaraldehyde solution within phosphate-buffered saline at pH 7.4 for a minimum of 2 hours. The samples were prepared as previously described (35, 36). The samples were subsequently mounted on double-sided carbon adhesive tape, gold-coated, and analyzed using a scanning electron microscope (Joel, JSM-5400LV, Tokyo 1993, Japan) at Assiut University’s Electron Microscopy Unit to assess the surface morphological alterations (37).

### 2.13 Statistical analysis

The chi-square test was utilized to analyze the prevalence and associated risk factors concerning the sex and age of the studied animals. The *in vitro* investigation results were processed utilizing SPSS software (version 20). Data are presented as mean ± standard deviation (SD), and differences between experimental groups were evaluated using a one-way ANOVA, with a significance threshold of *p* < 0.05 (38). For multiple group comparisons, *post hoc* analysis was performed, specifically utilizing the LSD and Duncan tests.

## 3 Results

### 3.1 Tick infestation and piroplasm infections

Out of 130 cattle examined, 61 (46.9%) were found to be infested with ticks. Analysis of thin blood films from these infested animals revealed an infection rate of 81.9% for blood piroplasm. The identified piroplasm infections included two species: *Theileria* and *Babesia*. As shown in Table 1, the chi-square test for *Theileria* infection showed a value of 3.6885 with a *p* = 0.05479, which is marginally significant, suggesting a potential association between *Theileria* infection and tick infestation. In contrast, the chi-square test for *Babesia* infection revealed a highly significant chi-square value of 22.443 with a *p =* 0.00002, indicating a strong association between *Babesia* infection and tick infestation.

**Table 1:** Associations between piroplasm infections and ticks.

Piropalsm	Infection status	Animals infested with ticks (61)	Percentage (%)	*χ*^2^	*p*-value
*Theileria* sp.	Non_infected	23	37.7	3.6885	0.05479
	Infected	38	62.3		
*Babesia* sp.	Non_infected	49	80.3	22.443	0.00002
	Infected	12	19.7		

Chi-square analysis was used to detect the association between tick infestation and the presence of *Theileria* and *Babesia* infection. A statistically significant difference (*p* < 0.05).

### 3.2 Molecular identification of positive blood samples

#### 3.2.1 Theileria annulata

**Figure 1:**
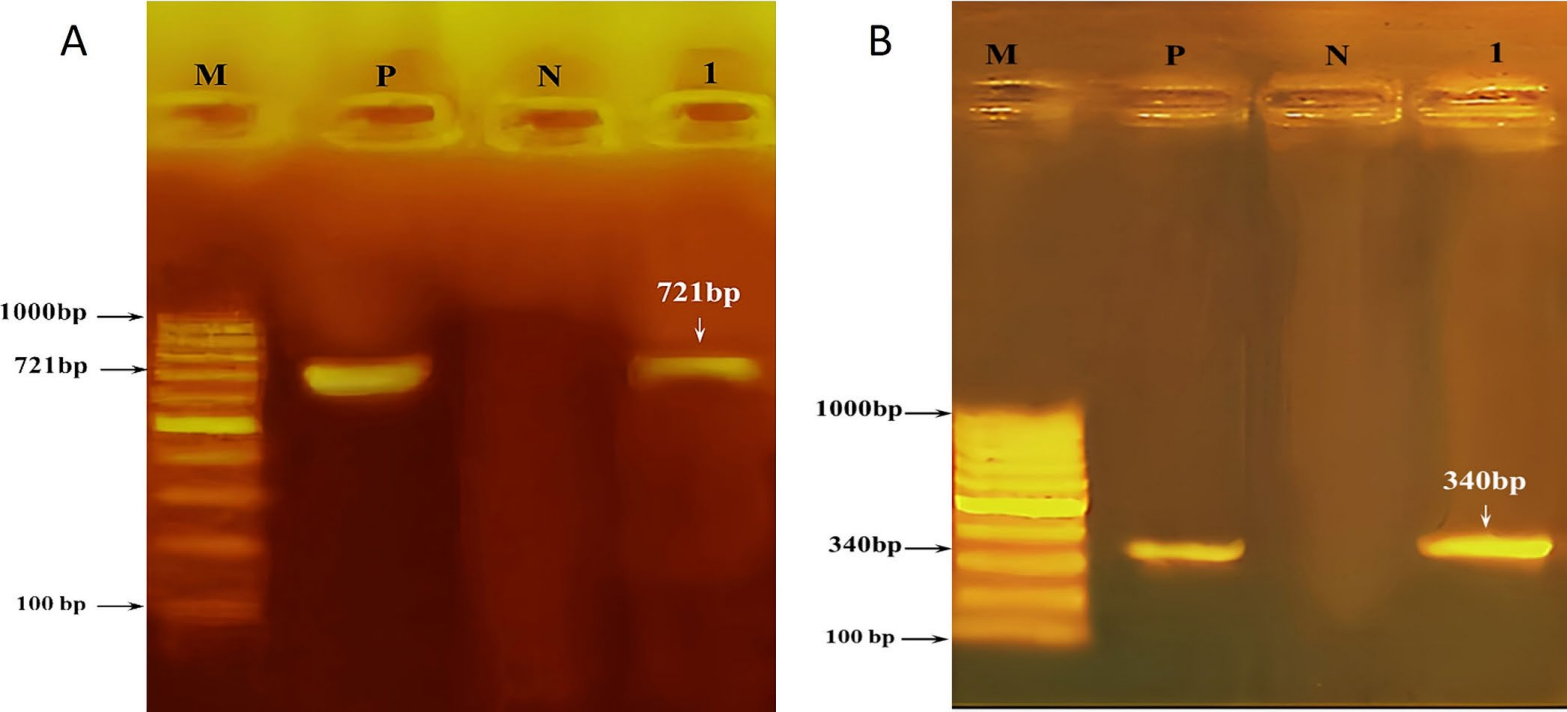
Agarose (1.5%) gel showing **(A)** PCR products (721 bp) of amplified *Theileria annulata* samples. **(B)** PCR products (340 bp) of amplified *Babesia bigemina*. Lane (M) is the DNA size marker, lane (P) is the positive control, lane (N) is the negative control, and lane (1) is the positive sample.

**Figure 2:**
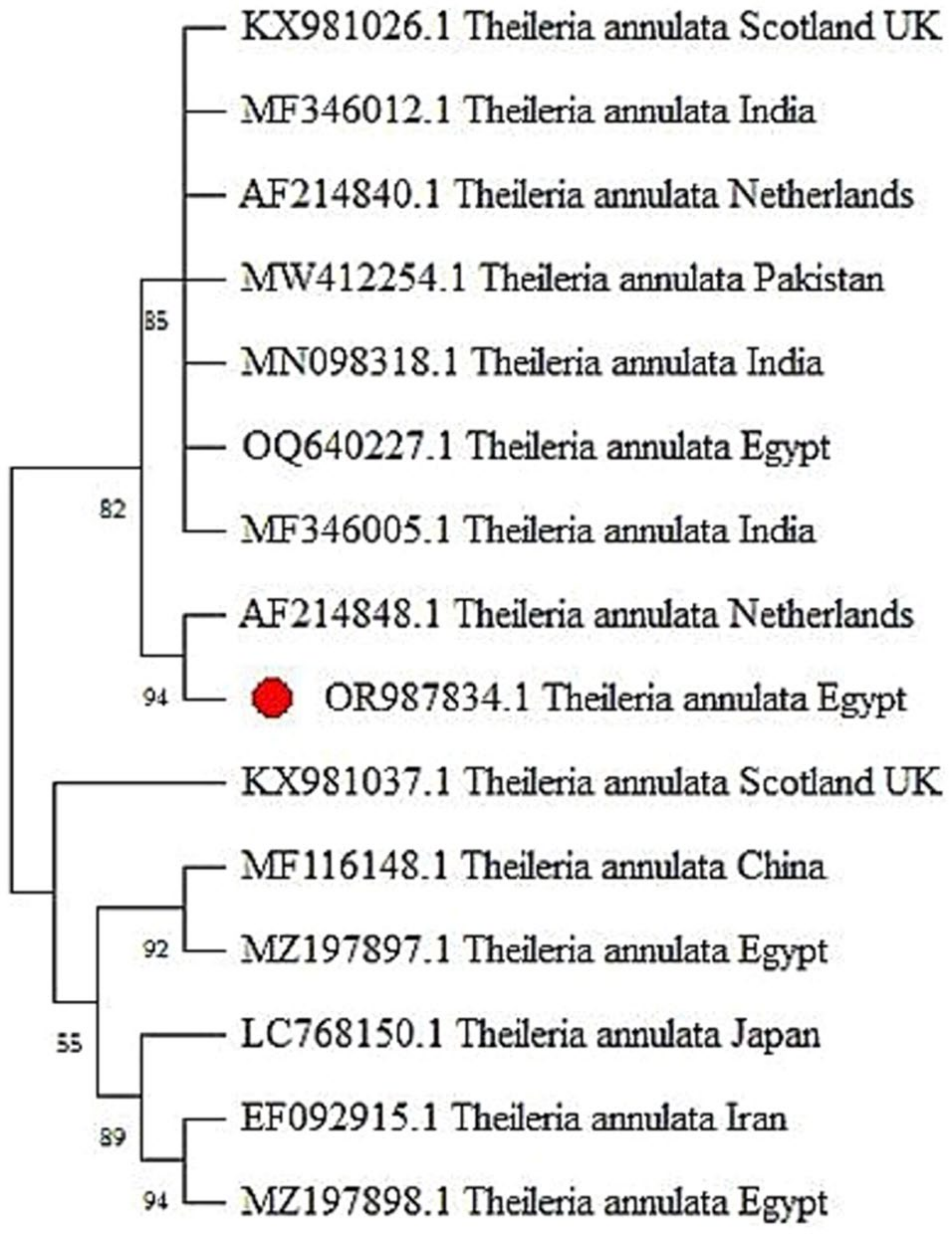
The phylogenetic tree generated from tams1 gene sequences, revealing evident clades that define distinct genetic lineages within *Theileria annulata*.

The PCR method was utilized to amplify the *Theileria annulata* tams1 gene, resulting in the identification of 721 bp bands observed under UV light on agarose gel (1.5%) (see Figure 1A). Sequencing of the resulted PCR product confirmed the affiliation of our sample with *Theileria annulata*. The sequence has been deposited in GenBank under the accession number OR987834. The phylogenetic relationship between the Tams1 nucleotide sequences of our isolate and 14 reference isolates of *Theileria annulata* is illustrated in Figure 2. This analysis provides insights into the genetic diversity and geographical distribution of the detected pathogen. The well-supported clade structure indicates that geographical factors significantly influence genetic variation among the isolates. Notably, there is a distinct clustering of isolates from Scotland, India, the Netherlands, Pakistan, and Egypt, highlighting the impact of geographical origins on the genetic makeup of these parasites. There is a robust subgroup, including the Egyptian isolate (OR987834.1) and the isolate from the Netherlands (AF214848.1).

#### 3.2.2 Babesia bigemina

**Figure 3:**
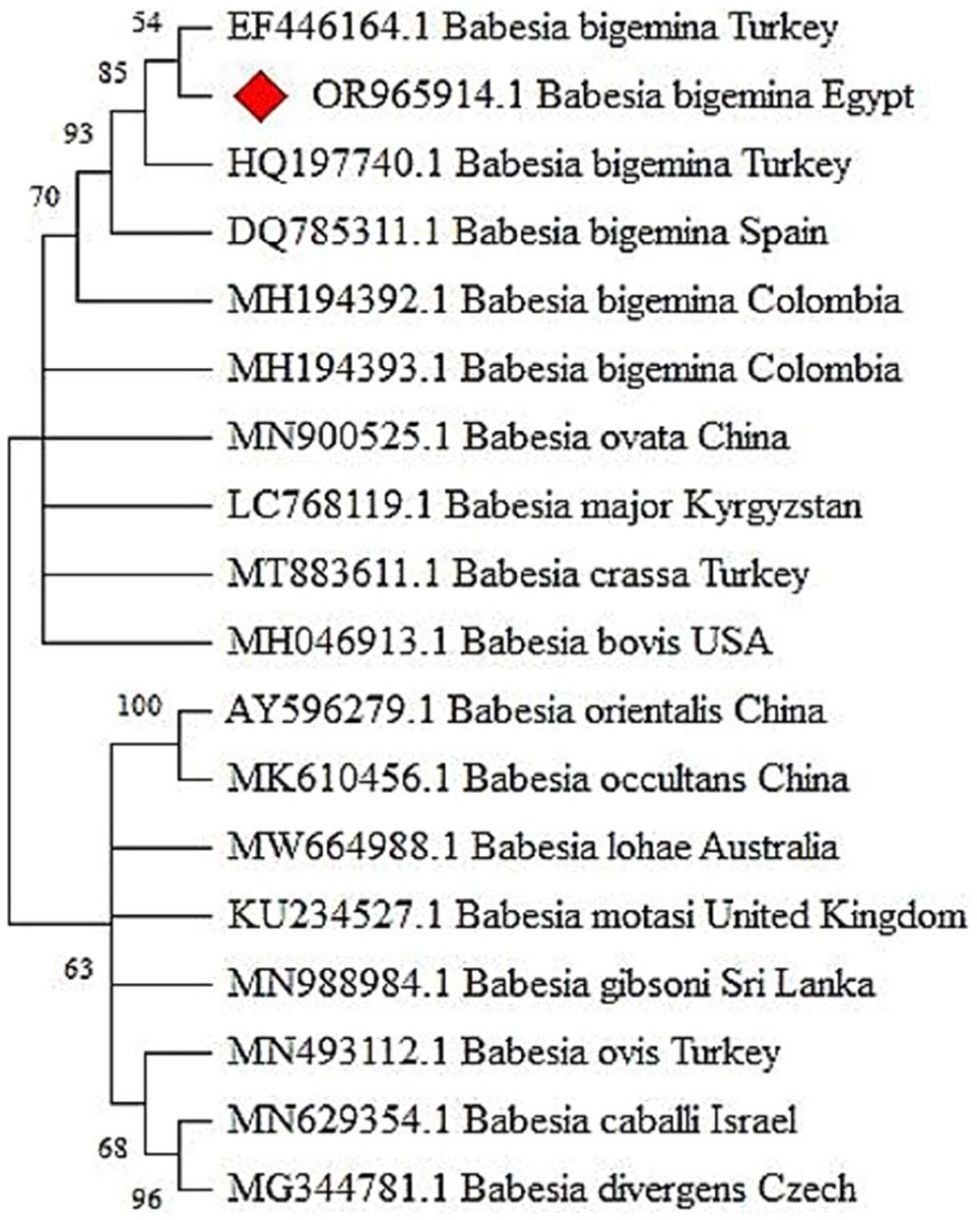
The phylogenetic tree constructed from the 18S rRNA gene sequences, unveils clear clades that delineate distinct genetic lineages of *Babesia bigemina*.

The PCR method was used to amplify *Babesia* 18S rRNA, resulting in the detection of 340 bp bands visible under UV light on agarose gel 1.5% (Figure 1B). Sequencing of the resulted PCR product confirmed that our sample is affiliated with *Babesia bigemina*. The corresponding sequence has been cataloged in GenBank under the accession number OR965914. The phylogenetic relationship between our isolate (18S rRNA nucleotide sequences) and 17 reference strains of *Babesia* species is shown in Figure 3. The well-supported clade includes the newly identified *Babesia bigemina* isolates from Egypt (OR965914.1) and the isolates from Turkey (HQ197740.1 and EF446164.1). In addition, the distinct subgroup formed by the *B. bigemina* Colombian isolates (MH194393.1 and MH194392.1) shows regional genetic diversity within this species. The phylogenetic tree further shows distinct differences between various *Babesia* species. *Babesia ovata* and *Babesia major* form a separate clade, while *Babesia crassa* and *Babesia bovis* group together. There is strong support for the clade containing *Babesia orientalis* and *Babesia occultans* isolates from China.

### 3.3 Hemolymph examination

**Figure 4:**
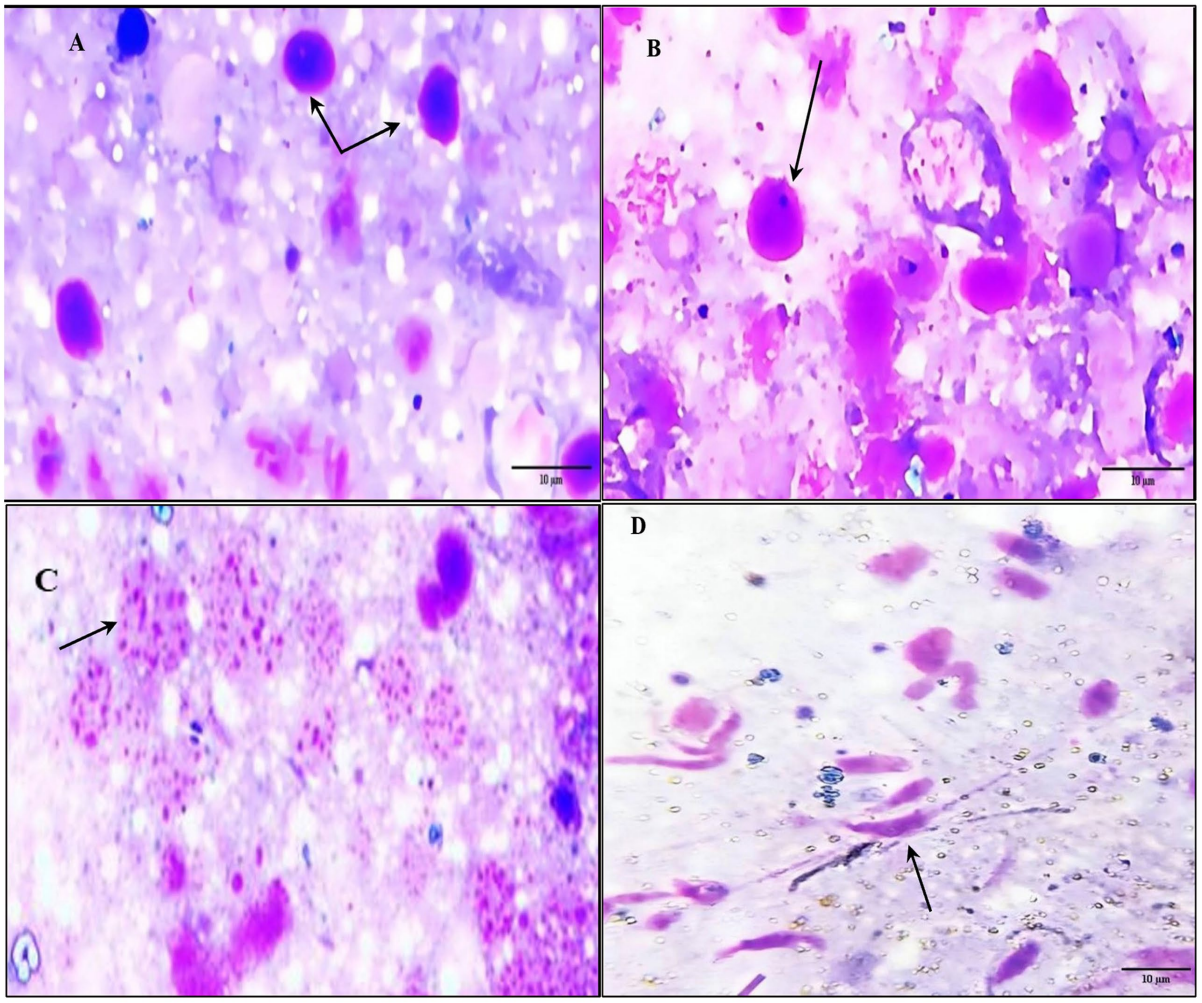
Photomicrograph showing different morphological characterization of hemolymph cells and piroplasm ookinetes in the infected samples. **(A)** Prohaemocyte (arrow). **(B)** Plasmatocytes (arrow). **(C)** Spherule (arrow). **(D)** Infected hemolymph containing piroplasm ookinetes (banana-shaped) with curved or semi-curved tails (arrow).

By light microscope, the tick hemolymph appeared to contain three main groups of cells (hemocytes), which can be classified into (prohemocytes, plasmatocytes, and spherulocytes) based on their structural characteristics. Prohemocytes are small and rounded with a high nuclear-cytoplasmic ratio and deeply basophilic cytoplasm. They have a round, single nucleus with condensed homogeneous chromatin. Plasmatocytes are round or ovoid, with a single nucleus. The chromatin is punctate or granular, and the nucleus is usually eccentric in position. Finally, spherulocytes are frequently large, ovoid, or round cells with cytoplasm containing characteristic purple staining clusters of spherules. These spherules are so dense that the cytoplasm and the nucleus are obscured. The infected hemolymph contains ookinetes of piroplasms, which are banana-shaped with curved or semi-curved tails. These ookinetes have an anteriorly positioned nucleus, typically located in the middle of the cell (Figure 4).

### 3.4 *Chrysanthemum* extract: total phenolic acid content

**Figure 5:**
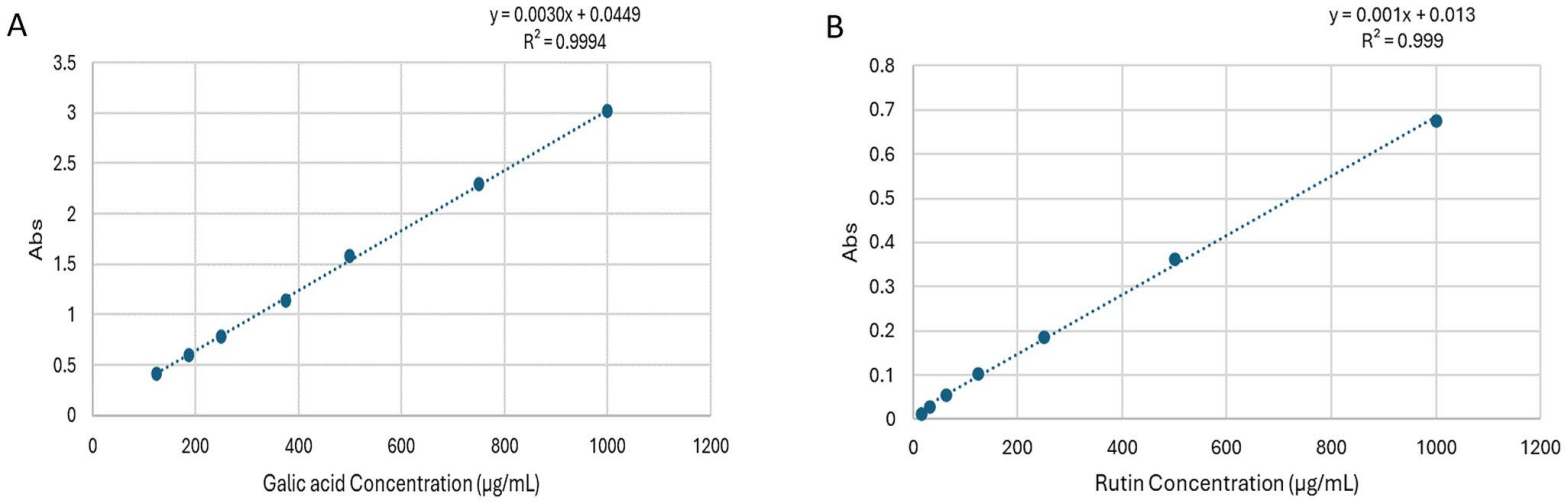
**(A)** Standard calibration curve showing absorbance versus gallic acid concentration for determining the total phenolic content of *Chrysanthemum* extract. **(B)** Standard calibration curve showing absorbance versus rutin concentration for determining the total phenolic content of *Chrysanthemum* extract.

The analysis of the total phenolic content within the hydro-methanolic extract was accomplished by referencing the gallic acid calibration curve (Figure 5A). The analysis revealed a substantial presence of phenolic compounds, with a measured content of 20.204 ± 1.75 μg gallic acid equivalent (GAE) per 1 mg of dry extract.

### 3.5 *Chrysanthemum* extract: total flavonoid content

The assessment of total flavonoid content in *Dendranthema grandiflora* aerial parts extract revealed noteworthy findings. Using the curve rutin calibration curve (Figure 5B), the flavonoid level was quantified, showcasing a significant flavonoid content of 34.25 ± 2.55 μg rutin equivalent (RE) per 1 mg of dry extract.

### 3.6 Acaricidal potential of *Chrysanthemum* extract and neem oil on *Rhipicephalus annulatus* adult stage

*Chrysanthemum* extract was tested at 0.5 mg/mL, 0.25 mg/mL, and 0.125 mg/mL. The acaricidal effect was increased over time, with concentrations of 0.5 mg/mL and 0.25 mg/mL showing the most pronounced effects. These concentrations induced tick mortality within the first 3 h of application, with mortality percentages of 73.3 and 60%, respectively, reaching complete mortality after 24 h. Phoxim caused mortality in all ticks within 12 h. Significant differences were observed between all concentrations of *Chrysanthemum* extract and the control group (*p* < 0.001). No significant differences were observed between different concentrations at various time points as shown in Table 2.

**Table 2:** Acaricidal effect of *Chrysanthemum* extract on *R. annulatus* adult mortality.

	Time (hours)					
	3 h	6 h	12 h	24 h	48 h	72 h
*Chrysanthemum* (0.5 mg/mL)	73.3 ± 11.5^aA^	80 ± 20^aA^	86.7 ± 11.5^abA^	100 ± 0^cA^	100 ± 0^cA^	100 ± 0^cA^
*Chrysanthemum* (0.25 mg/mL)	60 ± 20^aA^	73.3 ± 11.5^abA^	86.7 ± 11.5^bA^	100 ± 0^cA^	100 ± 0^cA^	100 ± 0^cA^
*Chrysanthemum* (0.125 mg/mL)	53.3 ± 11.5^aA^	60 ± 0^aA^	86.7 ± 11.5^bA^	86.7 ± 11.5^bA^	100 ± 0^cA^	100 ± 0^cA^
Phoxim	0^Ab^	60 ± 0^bA^	100 ± 0^cA^	100 ± 0^cA^	100 ± 0^cA^	100 ± 0^cA^
Control negative	0^B^	0^B^	0^B^	0^B^	0^B^	0^B^

Small superscript letters denote a significant difference (*p* < 0.05) in the mortality rate during the incubation periods in the same treated group (horizontal comparison). Capital superscript letters denote a significant difference (*p* < 0.05) in the mortality rate of the different treated groups in the same incubation period (vertical comparison).

Neem oil was evaluated at concentrations of 20 mg/L, 15 mg/L, and 10 mg/L. Significant differences were observed between the high and low concentrations of neem oil. The mortality rate increased over time, with concentrations of 20 mg/L and 15 mg/L exhibiting the most significant effects. These concentrations induced noticeable changes and caused tick mortality within the first 3 h of application, with mortality rates of 60%. Complete mortality was achieved after 24 h. Conversely, the minimal effect was observed in the lower concentration10 mg/L, resulted in only 13.3% mortality rate within the first 3 h, and achieved complete mortality after 72 h of treatment. Significant differences were observed between all concentrations of neem oil and the control group (*p* < 0.001). At various time points, significant differences were also noted between the high concentrations (20 mg/L and 15 mg/L) and the low concentrations (10 mg/L) as shown in Table 3.

**Table 3:** Acaricidal effect of neem oil on *R. annulatus* adult mortality.

Treatment	Time (hours)					
	3 h	6 h	12 h	24 h	48 h	72 h
Neem (20 mg/L)	60 ± 0^aA^	80 ± 0^bA^	86.7 ± 11.5^bA^	100 ± 0^cA^	100 ± 0^cA^	100 ± 0^cA^
Neem (15 mg/L)	60 ± 0^aA^	66.7 ± 11.5^aB^	80 ± 0^bA^	100 ± 0^cA^	100 ± 0^cA^	100 ± 0^cA^
Neem (10 mg/L)	13.3 ± 11.5^aB^	33.3 ± 11.5^bC^	33.3 ± 11.5^bB^	86.6 ± 11.5^cB^	86.6 ± 11.5^cB^	100 ± 0^cA^
Phoxim	0^aC^	60 ± 0^bB^	100 ± 0^cC^	100 ± 0^cA^	100 ± 0^cA^	100 ± 0^cA^
Control negative	0^C^	0^D^	0^D^	0^C^	0^C^	0^B^

Small superscript letters denote a significant difference (*p* < 0.05) in the mortality rate during the incubation periods in the same treated group (horizontal comparison). Capital superscript letters denote a significant difference (*p* < 0.05) in the mortality rate of the different treated groups in the same incubation period (vertical comparison).

### 3.7 Statistical correlation between high concentrations of *Chrysanthemum* extract (0.5 mg/mL) and neem oil (20 mg/L) with phoxim at various time intervals

**Figure 6:**
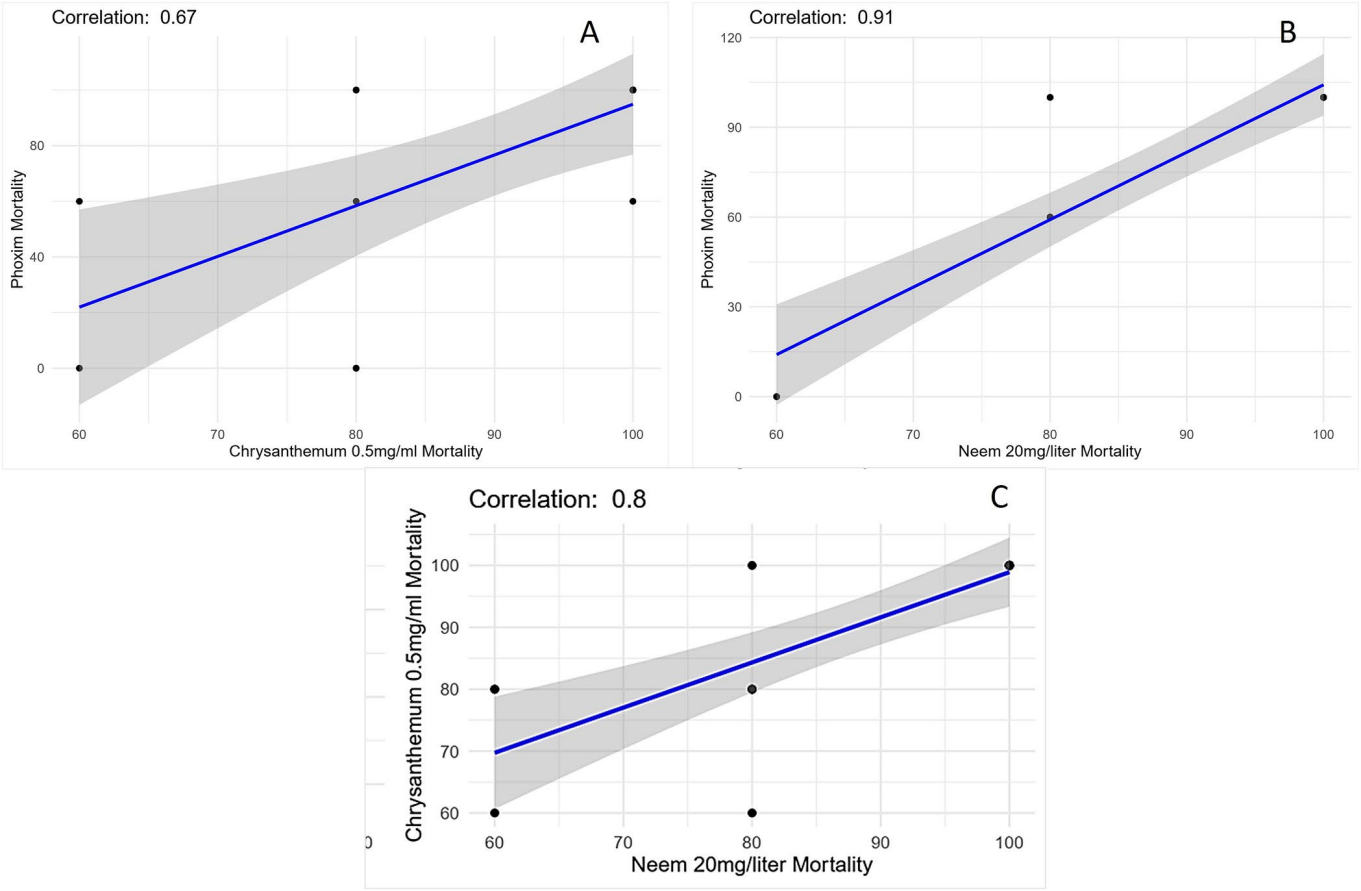
**(A)** Scatter plot illustrating the relationship between *Chrysanthemum* extract 0.5 mg/mL mortality and phoxim mortality with a moderate positive correlation (*r* = 0.67). **(B)** Scatter plot showing the relationship between neem oil 20 mg/L mortality and phoxim mortality with a strong positive correlation (*r* = 0.91). **(C)** Scatter plot showing the relationship between neem oil 20 mg/L mortality and *Chrysanthemum* extract 0.5 mg/mL mortality with a positive correlation (*r* = 0.8). The blue line represents the best-fit linear regression line, and the shaded gray area indicates the confidence interval.

The scatter plot shows the relationship between *Chrysanthemum* extract (0.5 mg/mL) and phoxim mortality rate (Figure 6A), the correlation coefficient is 0.67, which indicates a moderate positive linear relationship between *Chrysanthemum* extract and phoxim mortality rate, suggesting that higher mortality rates due to *Chrysanthemum* are associated with higher mortality rates due to phoxim. The correlation coefficient is 0.91 between neem oil (20 mg/L) and phoxim mortality rate, indicating a very strong positive linear relationship. As the mortality rate due to neem increases, the mortality rate due to phoxim also tends to increase. The plot contains data points representing pairs of mortality rates for neem and phoxim, most of which lie close to the fitted linear regression line, showing the data trend. Some data points lie further from the line and maybe outliers (Figure 6B). The scatter plot also demonstrates a strong positive correlation (*r* = 0.8) between neem (20 mg/L) and *Chrysanthemum* (0.5 mg/mL) treatments, indicating that as neem’s efficacy increases, *Chrysanthemum*’s mortality rate also rises as shown in Figure 6C.

### 3.8 Evaluation of the acaricidal effect using SEM

**Figure 7:**
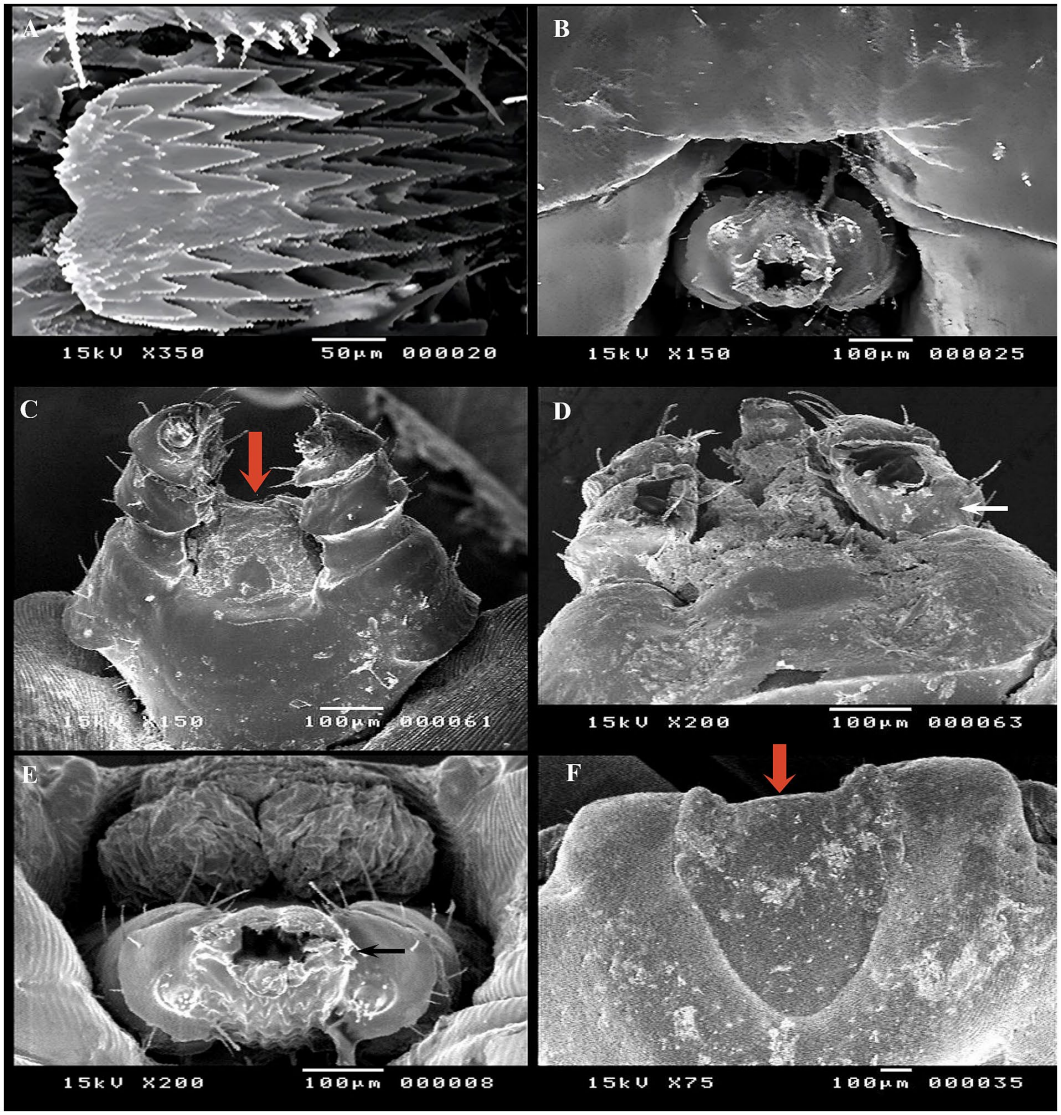
Acaricidal effects of the different groups on capitulum of adult female *R. annulatus* ticks. **(A)** Control group showing intact capitulum structure. **(B)** Phoxim-treated group with complete capitulum degeneration. **(C)** Neem oil-treated group displaying complete hypostome destruction. **(D)** Neem oil-treated group with damaged capitulum. **(E)** *Chrysanthemum* extract-treated group showing severe mouthpart damage. **(F)** *Chrysanthemum* extract-treated group with hidden capitulum.

**Figure 8:**
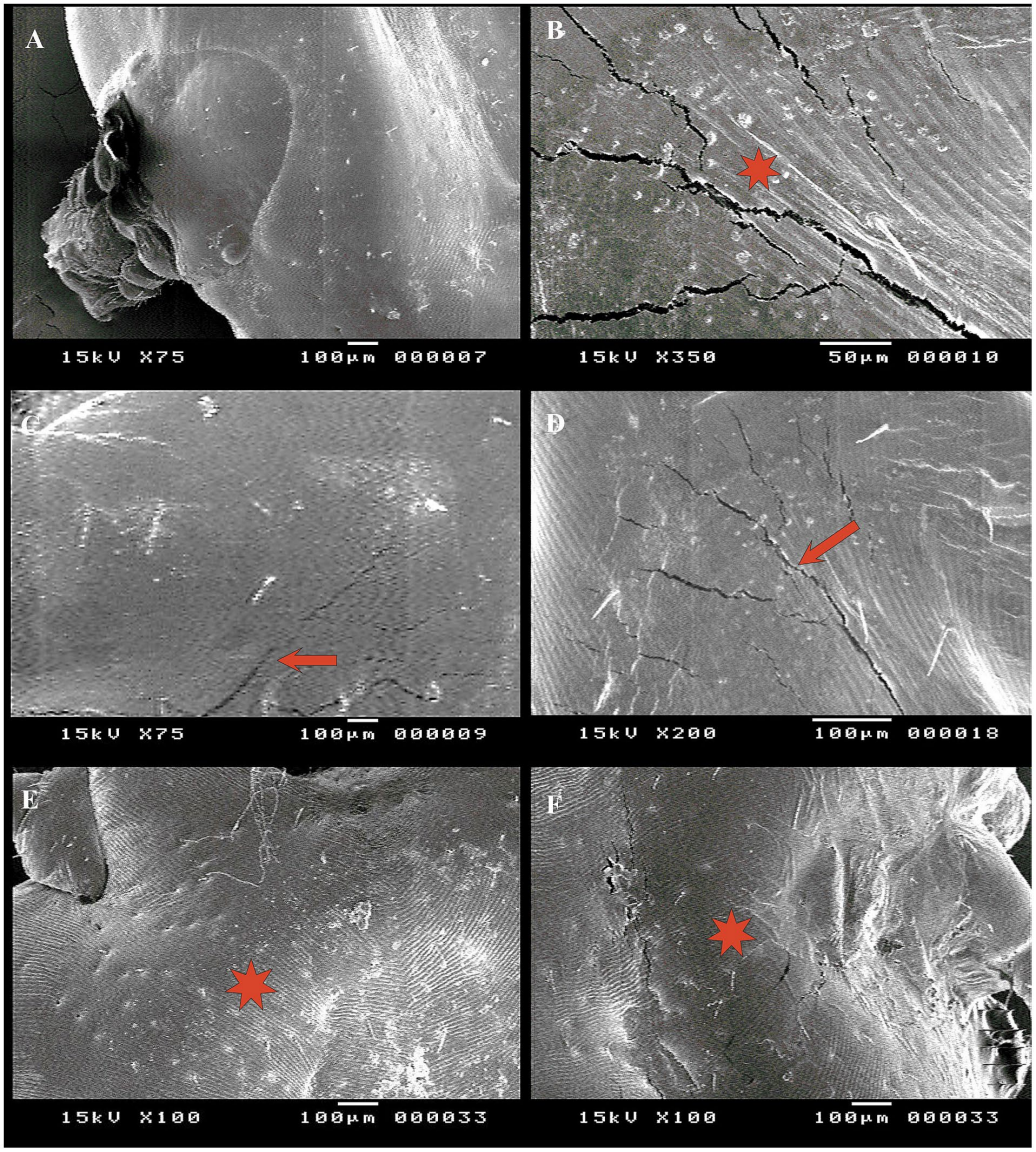
Acaricidal effects of the different groups on dorsal surface of adult female *R. annulatus* ticks. **(A)** Control group with intact anterior part. **(B)** Phoxim-treated group displaying cuticle cracks. **(C,D)** Neem oil-treated group showing cuticle cracking. **(E)** *Chrysanthemum* extract-treated group showing pores in the cuticle. **(F)** *Chrysanthemum* extract-treated group with cuticle cracks.

**Figure 9:**
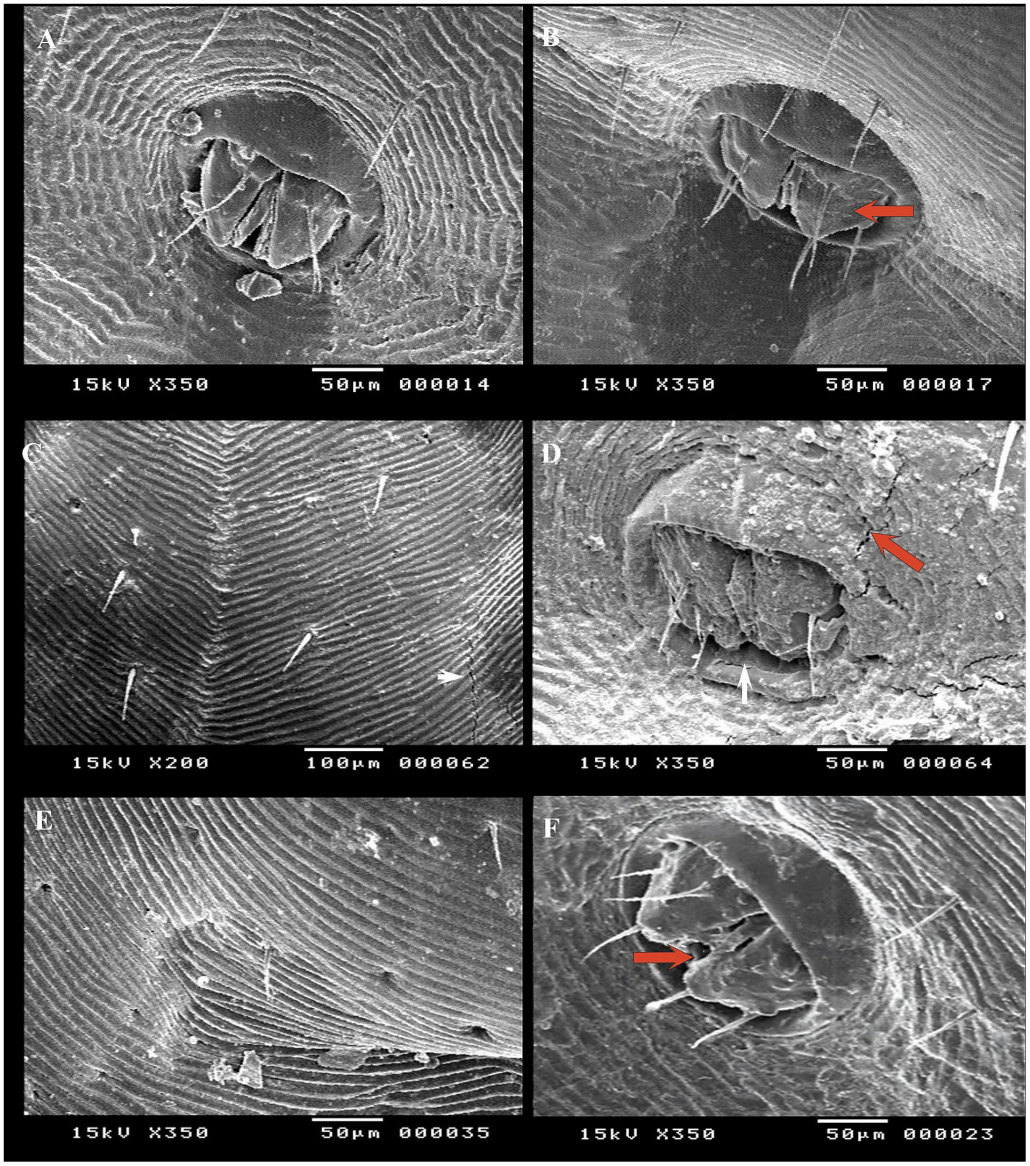
Acaricidal effects of the different groups on the ventral surface of adult female *R. annulatus* ticks. **(A)** Control group maintaining normal appearance. **(B)** Phoxim-treated group with retracted anal region. **(C)** Neem oil-treated group showing the typical distribution of striations with some pores. **(D)** Neem oil-treated group showing destruction and cracks around the anal region. **(E)** *Chrysanthemum* extract-treated group with pore formation. **(F)** *Chrysanthemum* extract-treated group with wrinkling and slight degeneration of the anal region.

SEM was conducted to observe surface changes in various parts of adult female *R. annulatus* specifically the capitulum, dorsal surface, and ventral surface following treatments with neem oil (20 mg/L), *Chrysanthemum* extract (0.5 mg/mL), and phoxim (1 mL/L). In the control negative group, the capitulum maintained its intact structure (Figure 7A). In contrast, the phoxim-treated group showed complete degeneration of the capitulum (Figure 7B). Neem oil treatment caused significant damage, destroying both the hypostome and the capitulum (Figures 7C,D). Similarly, *Chrysanthemum* extract caused severe damage, with complete degeneration of the mouthparts and the capitulum becoming obscured (Figures 7E,F). Upon examining the dorsal surface, the control group (Figure 8A) exhibited an intact anterior region. In contrast, the phoxim-treated group (Figure 8B) revealed numerous cracks in the cuticle. The neem oil treatment resulted in cracking of the cuticle (Figures 8C,D). However, *Chrysanthemum* extract caused more severe damage, featuring visible pores and significant cracks in the cuticle (Figures 8E,F). On the ventral surface around the anal region, the control group exhibited a normal appearance (Figure 9A), while the phoxim-treated group showed a retracted anal region (Figure 9B). However, treatment with neem oil resulted in notable changes, including retraction of the anal region, destruction accompanied by cracks and typical distribution of striations in some area with pores (Figures 9C,D), In contrast, *Chrysanthemum* extract caused wrinkling of the cuticle, leading to the formation of pores or holes (Figure 9E) and a disrupted striation pattern, along with slight degeneration of the anal region (Figure 9F).

## 4 Discussion

### 4.1 Prevalence of tick-borne piroplasm infection

This study investigated the prevalence of tick-borne piroplasms infection (*Babesia* and *Theileria*) in cattle and explored the efficacy of *Chrysanthemum* extract and neem oil as potential natural acaricides. Our results showed significant associations between tick infestation and piroplasm infections, with *Theileria* infections being more prevalent (62.3%) than *Babesia* infections (19.7%). These findings align with previous studies in Egypt, where *Babesia* and *Theileria* infections were reported in 11.16 and 10.25% of cattle, respectively, with *B. bigemina* and *T. annulata* being the most common species (39). However, high prevalence of *Theileria* than *Babesia* infection were reported by Prado et al. (40) and Hossain et al. (41), whereas El-Dakhly et al. (42) reported that prevalence of *Theileria* infection was (9.31%) in cattle in El-Wadi El-Gadid province. Additionally, the prevalence of *Theileria* infection was 8.3% in Sudan (43) and Bangladesh (41). In our study, *Theileria* sp. has a higher prevalence than *Babesia* sp. due to a variety of factors, including the availability of suitable tick vectors, reservoirs, and amplification hosts. These factors all contribute to the spread and persistence of *Theileria* in each ecosystem (44), which lower than those reported by El-Metenawy (45) in Saudi Arabia (76.5%) and Al-Emarah et al. (46) in Iraq (69.43%). This study contributes valuable insights into the dynamics of tick-borne diseases and enhances our understanding of their epidemiology (47).

### 4.2 Molecular characterization of piroplasm infection

To identify piroplasm species, the Tams1 gene for *Theileria* and the 18S rRNA gene for *Babesia* were amplified and sequenced. Phylogenetic analysis revealed genetic diversity within *T. annulata* and *B. bigemina* isolates, suggesting a need for region-specific control measures. Phylogenetic analysis of *T. annulata* revealed significant genetic diversity influenced by geography, with isolates from different regions such as Scotland, India, the Netherlands, Pakistan, and Egypt forming distinct clusters. There are multiple Egyptian isolates in different clades highlighting high genetic variability within the local population aligning with another study in upper Egypt that estimated the presence of *T. annulata* infection in cattle (48). On the other hand, the phylogenetic tree of *B. bigemina* showed a close genetic relationship between the Egyptian isolate and those from Turkey. This indicates regional genetic diversity, with distinct clades for various *Babesia* species and clear evolutionary relationships observed.

In this study, morphological characteristics of piroplasm ookinetes were examined in tick hemolymph. The presence of ookinetes, crucial for active infections, was confirmed using their distinct banana shape, curved tails, and central nucleus. These results align with findings from Martínez-García et al. (49), and are key to recognizing piroplasm ookinetes, which play a crucial role in active infections (49). Our results contribute to the broader understanding of piroplasm morphology and the role of ticks as vectors of hemoprotozoan parasites, offering valuable insights into infection dynamics.

### 4.3 Acaricidal potential of *Chrysanthemum* extract and neem oil on *Rhipicephalus annulatus* adult stage

Integrating botanical insecticides into tick management programs can contribute to a holistic approach to animal health, addressing ectoparasite control and overall well-being. Given the growing resistance of ticks to commercial acaricides, this study evaluated *Chrysanthemum* extract and neem oil as potential natural eco-friendly alternatives. Our results demonstrate that both extracts had significant acaricidal activity against ticks. *Chrysanthemum* extract, particularly at high concentrations (0.5 mg/mL), showed high efficacy in inducing tick mortality, resulting in 73.3% mortality within the first 3 h, reaching complete mortality at 24 h. Our findings aligned with a study that demonstrated that the effects of *Chrysanthemum roseum* extracts achieved 100% mortality against *Rhipicephalus microplus* at a 5% concentration (50). In this study, the statistical analysis showed a moderate correlation between *Chrysanthemum* extract (0.5 mg/mL) and phoxim mortality rates with significant differences between all concentrations of *Chrysanthemum* extract and the control group. Organic repellent utilizing *Chrysanthemum* oil on 5 mL, resulted in the killing of insects such as ants, cockroaches, and flies after 2.81 min (51). Notably, pyrethrins, derived from Pyrethrum, laid the foundation for the synthesis of more potent synthetic pyrethroids. The insecticidal activity of Pyrethrum, with its rapid biodegradation and its relatively low mammalian toxicity, makes it one of the most widely used non-synthetic insecticides in certified organic agriculture (17). In the present study, the *Chrysanthemum* extract has abundant phenolic and flavonoid contents, which contribute to its potential bioactivity and therapeutic value. In addition, the high flavonoid content in the *Chrysanthemum* extract suggests potential therapeutic value and health benefits. Phenolic compounds are well-known for their antioxidant properties, anti-inflammatory, antimicrobial, and anti-carcinogenic activities (52–54). Flavonoids are renowned for their diverse pharmacological effects, including antioxidant and anti-inflammatory properties, and anti-cancer effects, among others (55).

Neem oil, at concentrations of 20, 15, and 10 mg/L, exhibited significant acaricidal effects. The highest concentration (20 mg/L) resulted in 60% mortality within the first 3 h and reaching complete mortality after 24 h. Significant differences were observed between all concentrations of neem oil and the control group, as well as between higher and lower concentrations of neem oil at different time points. These results are consistent with the study by Gareh et al. (33), which revealed that 100% mortality of adult ticks on the 1st day post-treatment. The correlation analysis between the high concentration of neem oil (20 mg/L) and phoxim mortality rates showed a strong positive linear relationship, while the mortality rate due to neem oil increased, the phoxim mortality rate also increased. This indicates that *Chrysanthemum* and neem oil could be a powerful alternative to phoxim, as it has also shown insecticidal effects on numerous insect species, such as mosquitoes (56).

### 4.4 Detection the morphological alteration using SEM

SEM results confirmed that all treatments (*Chrysanthemum* extract, neem oil, and phoxim) caused notable damage to the tick cuticle, including wrinkling, pores, loss of striation, and cracks in some areas. The destruction of the hypostome was particularly evident when neem oil was used. This indicates that neem oil has the potential to interfere with the survival and reproduction of ticks. This effect may be due to the active components of neem, salannin, which have insect growth-regulating and antifeedant activity (33). The destruction of mouthparts with neem oil is similar to the effects noted with *Melia azedarach* and *Artemisia herba-alba* extracts on *Hyalomma dromedarii* (57). On the other hand, *Chrysanthemum* extract induced pronounced damage to the tick cuticle, marked by cracks and pores. Phoxim was effective in causing complete degeneration of the capitulum and extensive cuticle cracks. Also, cracks resulting from *Chrysanthemum* and phoxim were similar to those caused by *Cymbopogon citratus* oil on *Haemaphysalis longicornis* ticks (58). In the neem oil-treated group, the ventral surface and anal region of ticks showed retraction and destruction. In contrast, *Chrysanthemum* extract caused loss of striations and wrinkling with pore formation. These findings suggest that *Chrysanthemum* extract and neem oil demonstrate effective acaricidal effects similar to phoxim, confirming their potential as eco-friendly alternatives in integrated pest management strategies.

## 5 Conclusion

Our research highlights the strong connection between tick infestations and piroplasm infection in cattle, specifically *Babesia* species. Molecular identification confirmed the presence of *T. annulata* and *B. bigemina*. Phylogenetic analyses revealed significant genetic diversity influenced by geographical factors. *Chrysanthemum* extract, as well as neem oil, demonstrated high acaricidal efficacy, resulting in substantial mortality rates in *R. annulatus* ticks. Both treatments showed time-and dose-dependent effectiveness, causing severe morphological damage to the ticks, as demonstrated by SEM analysis. Overall, the findings support the use of *Chrysanthemum* extract and neem oil as effective natural alternatives for tick control, providing a sustainable solution for livestock health management.

## Data Availability

The datasets presented in this study can be found in online repositories. The names of the repository/repositories and accession number(s) can be found in the article/supplementary material.
